# PGE_2_ promotes macrophage recruitment and neovascularization in murine wet-type AMD models

**DOI:** 10.1186/s12964-022-00973-6

**Published:** 2022-10-13

**Authors:** Pengfei Zhan, Yuqing Cui, Yujuan Cao, Xun Bao, Meili Wu, Qian Yang, Jiahui Yang, Haohan Zheng, Jian Zou, Tianhua Xie, Jiping Cai, Yong Yao, Xiaolu Wang

**Affiliations:** 1 Department of Ophthalmology, The Affiliated Wuxi People’s Hospital of Nanjing Medical University, 299 Qingyang Road, Wuxi, 214023 Jiangsu People’s Republic of China; 2 Center of Clinical Research, The Affiliated Wuxi People’s Hospital of Nanjing Medical University, 299 Qingyang Road, Wuxi, 214023 Jiangsu People’s Republic of China; 3 Department of Ophthalmology, The Affiliated Wuxi No.2 People’s Hospital of Nanjing Medical University, Wuxi, 214023 Jiangsu People’s Republic of China

**Keywords:** PGE2, Neovascularization, Macrophage, AMD

## Abstract

Age-related macular degeneration (AMD), a progressive chronic disease of the central retina, is a leading cause of blindness worldwide. Activated macrophages recruited to the injured eyes greatly contribute to the pathogenesis of choroidal neovascularization (CNV) in exudative AMD (wet AMD). This study describes the effects of cyclooxygenase-2 (COX2)/prostaglandin E_2_ (PGE_2_) signalling on the macrophage activation and CNV formation of wet AMD. In a mouse model of laser-induced wet AMD, the mice received an intravitreal injection of celecoxib (a selective COX2 inhibitor). Optical coherence tomography (OCT), fundus fluorescein angiography (FFA), choroidal histology of the CNV lesions, and biochemical markers were assessed. The level of PGE_2_ expression was high in the laser-induced CNV lesions. Macrophage recruitment and CNV development were significantly less after celecoxib treatment. E-prostanoid1 receptor (EP_1_R)/protein kinase C (PKC) signalling was involved in M2 macrophage activation and interleukin-10 (IL-10) production of bone marrow-derived macrophages (BMDMs) in vitro. In addition, IL-10 was found to induce the proliferation and migration of human choroidal microvascular endothelial cells (HCECs). Thus, the PGE_2_/EP_1_R signalling network serves as a potential therapeutic target for CNV of the wet-type AMD.

**Graphical Abstract:**

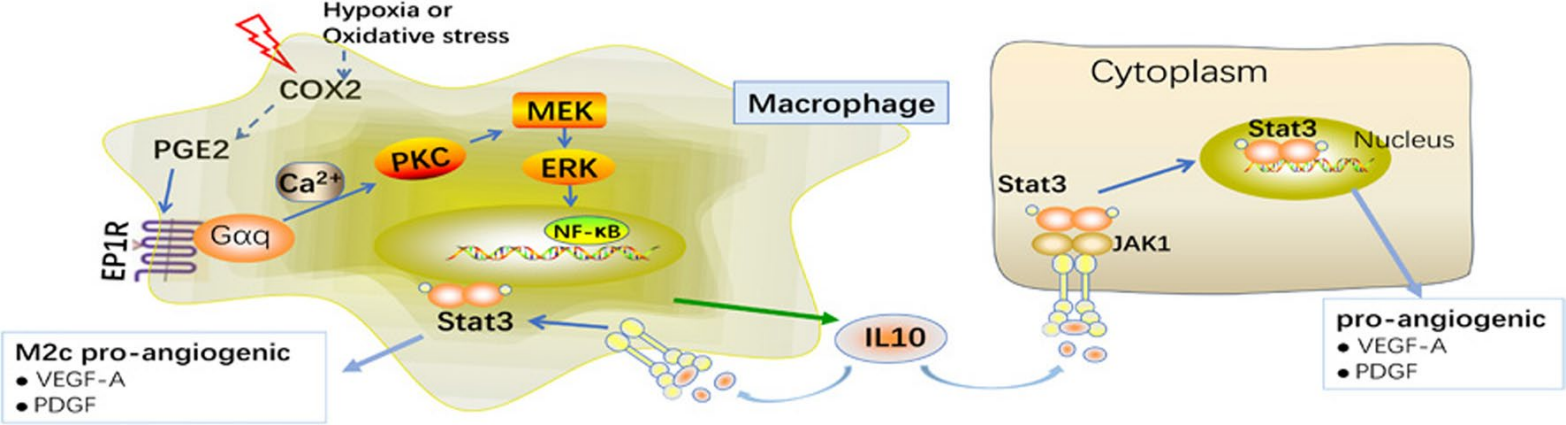

**Video abstract**

**Supplementary Information:**

The online version contains supplementary material available at 10.1186/s12964-022-00973-6.

## Background

Age-related macular degeneration (AMD) is a progressive chronic disease commonly occurring in individuals over 50 years of age with successive or simultaneous onset in both the eyes [1]. AMD results from the deterioration of the retina, particularly the retinal pigment epithelium (RPE), photoreceptor cell layer, and choroid in the macular area of the fundus [1]. It is categorized as either wet type (neovascular or exudative) or dry type (geographic atrophy) [2]. Wet AMD, a neovascular disease, is characterized by macular degeneration involving the formation of choroidal neovascularization (CNV) accompanied with fluid leakage from the choroidal neovessels [2]. With the progress of CNV, the retina is invaded, increasing oxidative stress, vascular leakage, and inflammation, thereby leading to haemorrhage and fibrosis of the retinal macula, loss of photoreceptor cells, and ultimately complete vision loss [3]. The main treatment strategy for wet AMD includes the use of anti-VEGF-A agents, however, such agents frequently require repeated intravitreal injection (IVI) [4]. Thus, there is an urgent need to devise novel interventional approaches.

CNV involves the infiltration of immune cells, such as macrophages, microglial cells, dendritic cells, and T cells, into the lesions [5]. In particular, macrophages are thought to be one of the main type of vascular-modifying immune cells that are attracted to the retina in CNV; therefore, an increase in the number of retinal macrophages serves as the hallmark of CNV [6]. Owing to the pro-inflammatory and proangiogenic properties of macrophages, inhibition of their migration to the retina has been shown to suppress CNV in a laser-induced mouse model of the disease [7]. Activated macrophages can be roughly classified as M1-type (pro-inflammatory/classically activated) or M2-type (anti-inflammatory/alternatively activated) [8]. M1 macrophages, regulated by pathogen-associated molecular patterns (e.g., lipopolysaccharide [LPS]) and inflammatory cytokines (e.g., Interferon *γ* [IFN-*γ*]) defend against the invading microbial pathogens such as bacteria, protozoa, and viruses [8]; whereas M2 macrophages are regulated by anti-inflammatory (e.g., TGF-*β* and interleukin-10 [IL-10]) and Th2 (e.g., IL-13 and IL-4) cytokines, glucocorticoids, immune complexes, and other factors and involved in various chronic inflammatory diseases such as allergies, obesity, parasite infections, and proangiogenic responses occurring in cancer and tissue remodelling and repair [8]. Several proteins are used as markers to distinguish between M1 and M2 macrophages. Inducible NOS (iNOS), costimulatory molecules (CD80 and CD86), and MHC class II agents are markers for M1 polarization [9]. Arginase-1, Fizz1, Ym1, IL-10, Mannose receptor (MR, CD206), macrophage galactose N-acetyl-galactosamine specific lectin, (Mgl, CD301), programmed cell death 1 ligand 2 (PD-L2, CD273), and PD-L1 (CD274) are useful markers for M2 polarization [9]. Polarization of monocytes/macrophages into M2 cells may be induced by various stimuli, such as IL-4 and/or IL-13 (M2a, alternatively activated macrophages); immune complexes and LPS (M2b); IL-10, prostaglandins, TGFβ or glucocorticoids (M2c, regulatory macrophages); adenosine and IL-6 (M2d, only in mice) [8, 9]. M2 macrophages promote wound healing and tissue remodelling and generate anti-inflammatory cytokines such as IL-10 and relatively low levels of TNF-*α* and IL-12 [9]. IL-4-induced M2a and IL-10-induced M2c macrophages have been demonstrated to promote angiogenesis in vitro as well as in vivo [10]. Moreover, the M2 macrophages recruited to the injured retina greatly contribute to the pathogenesis of CNV in wet AMD [11]; however, the molecular mechanisms underlying the recruitment of M2 macrophages are poorly understood.

Prostaglandin E_2_ (PGE_2_), a potent inflammatory mediator, is a crucial IL-1*β* inducer that causes fever [12]. PGE_2_ is biosynthesized from arachidonic acid by the cyclooxygenase enzyme and stimulates G-protein-coupled plasma membrane receptors (E-prostanoid 1, 2, 3 and 4 receptors [EP_1–4_Rs]), activating multiple signal transduction pathways leading to downstream responses [13]. EP_1_R mainly recruits the Gαq protein, upregulating the level of intracellular calcium and stimulating protein kinase C (PKC) signalling [14]. EP_2_R and EP_4_R bind to the Gαs protein and induce adenylate cyclase activation and upregulate cAMP/PKA signalling [14]. In contrast, EP_3_R couples with the Gαi protein and inactivates adenylate cyclase and inhibits the formation of intracellular cAMP [14]. Recent studies have shown that PGE_2_ promotes the polarization of M2c macrophages [15]. However, the molecular mechanism underlying the role of PGE_2_ in macrophage activation and CNV progression should be further clarified. Here, we show that PGE_2_ regulates macrophage activation and IL-10 secretion through the autocrine and paracrine pathways underlying pathological angiogenesis and vascular leakage in the mouse models of wet AMD. Moreover, the blockade of PGE_2_ signalling has been confirmed to strongly inhibit CNV.

## Materials and methods

### Animals

The mice were purchased from the laboratory animal centre of the Academy of Military Medical Sciences (Beijing, China) and housed under standard conditions (22.5 °C and 42.5% humidity, under a 12 h/12 h light–dark cycle, using heated wood chip litter as bedding material) in the SPF animal centre of Wuxi People’s Hospital Affiliated to Nanjing Medical University, and permitted ad libitum consumption of water. The animals were ventilated after being anaesthetized with a mixture of ketamine and xylazine and the effectiveness of the anaesthesia was monitored by observing the parameters like slow breathing, loss of muscular tone, and no response to surgical manipulation. The retina was then harvested for subsequent analyses. All the studies were conducted in accordance with the Guide for the Care and Use of Laboratory Animals (National Institutes of Health [NIH], Bethesda, MD, USA) and the ARVO Statement for the Use of Animals in the Ophthalmic and Vision Research. All the animal experiments fulfilled the requirements for humane animal care stated by the Nanjing Medical University.

### Laser-induced AMD model

The mice were anaesthetized, and the pupils were dilated by applying Cyclomydril (Alcon, Fort Worth, TX, USA). Using a 532-nm laser, a slit-lamp delivery system, and a sliding glass as a contact lens, 4 spots (laser power, 200 mW; exposure time, 100 ms; hole size, 75 μm) were placed into each eye at a distance of approximately 2 optic disc diameters from the optic nerve head. After the laser-induced injury, the clodronate group mice (> 20 weeks old) received an intravitreal injection with 2 μl/eye of 90 μg clodronate liposomes (including 16 μg of clodronic acid) mixed with 154 mM sodium chloride at a ratio of 1:1 or control liposomes using a 33-gauge needle. The eyes of a selective cyclooxygenase-2 (COX2) inhibitor celecoxib (Cayman Chemical Company #169,590–42-5) injection group were subjected to IVI of celecoxib (10 mM celecoxib dissolved in 154 mM sodium chloride at a ratio of 1:3), or DMSO (vehicle, 25%). In some cases, celecoxib was administered orally at a dose of 100 mg/kg/day. Celecoxib was dissolved in 100% ethanol (v/v) and mixed with the food. After the ethanol was completely volatilized, the food was fed to the mice daily. The control mice were fed with the volatilized ethanol-treated food daily.

### Retinal imaging

The animals were anaesthetized and the eyes were dilated and the Optical coherence tomography (OCT) images were obtained using an image-guided OCT system (Micron IV; Phoenix Research Labs, Pleasanton, CA, USA). The fundus fluorescein angiography (FFA) was performed using the Micron IV fundoscopy system (Phoenix Research Labs, Pleasanton, CA, USA). The anaesthetized animals with dilated pupils were intraperitoneally injected with 0.1 ml 0.2% sodium fluorescein (Fluorescite 10%; Alcon, Fort Worth, TX, USA).

### Flow cytometry

On day 7 after laser-induced CNV, mice were transcardially perfused with iced-cold PBS. After eyes were quickly removed, the retina and RPE-choroid-sclera complexes were cut into small pieces. The tissue was further mechanically dissociated by trituration and the suspension was applied to 30 um cell strainer. The single cells were pre-incubated with Fc-block followed by stained with FITC-conjugated anti-CD11b (#101,206, BioLegend), APC-conjugated anti-CD80 antibodies (#104,714, BioLegend), PE-conjugated anti-CD80 (#104,708), PE-conjugated anti-CD206 (#141,706, BioLegend) and/or APC-conjugated anti-CD206. Stained cells were processed using LSR-II cytometer (BD Biosciences), and the data were analysed using FlowJo Software (version 7.6.2).

### Cell culture and treatments

In our study, the general bone marrow-derived macrophages (BMDMs) were isolated from the C57BL/6 J mice. The isolation and culture of BMDM were performed as described [16]. Briefly, the animals were sacrificed by cervical dislocation and soaked in 75% ethanol. Then, the femurs and tibias were harvested and the bone marrow cells from all the bones were flushed out. After centrifuging for 5 min at 300 × g, the erythrocytes were eliminated using the red blood cell lysing buffer (Sigma-Aldrich, St. Louis, MO, USA). The remaining cells were seeded in the plates and incubated in a complete medium with 50 mg/ml recombinant mouse macrophage colony-stimulating factor (M-CSF, R&D Systems #416-ML) for 7 days to form the proliferative nonactivated cells (also named M0 macrophages).

Besides, the cells grown in DMEM were incubated with PGE_2_ (1 μM or 5 μM; Cayman Chemical Company #14,010), H_2_O_2_ (100, 200, 400, 800 μM) or recombinant murine IL-4 (20 ng/mL, PeproTech #214–14) or LPS (10 ng/ml #L4391) or recombinant human IL 10 (25, 50 ng/ml, PeproTech #200–10) with different time points. In some experiments, selective celecoxib (10 μM), 17-PT-PGE_2_ (an EP_1_R agonist, 10 μM), Butaprost (an EP_2_R agonist, 10 μM #13,740), Sulprostone (an EP_3_R agonist, 10 μM #14,765), Cay10598 (an EP_4_R agonist, 10 μM #13,281), H89 (a PKA inhibitor, 5 μM #10,010,556), LY294002 (a PKB inhibitor, 5 μM #70,920), Bis1 (a PKC inhibitor, 5 μM) obtained from the Cayman Chemical Company (Ann Arbor, MI, USA) were added to cells.

### The haematoxylin and eosin (H&E) staining

The mice were anaesthetised, and their eyes were dissected and fixed in 4% paraformaldehyde (wt./vol.) overnight. The retina and the RPE-choroid-sclera complexes were dehydrated in a graded ethanol series and embedded in paraffin. For H&E staining, the 5-μ-thick sections were taken along the vertical meridian. The digital images of H&E staining were observed under an Olympus BX-51 light microscope (Olympus, Tokyo, Japan).

### Immunocytostaining

The standard immunofluorescence analysis was performed to indicate the expression of the protein, followed by secondary antibodies (Thermo Fisher Scientific, CA, USA) in the cells or tissue sections, as previously described [17].

The volume of the CNV lesions was measured in the choroidal flat mounts after injury. The anterior segment and retina were removed from the eyecup after fixation in 4% paraformaldehyde in PBS. The remaining RPE-choroid complex was dehydrated in methanol and stained with 7 μg/ml fluorescein-labelled IB4 (Thermo Fisher Scientific #I21411). After relaxing radial incisions, this complex was flat-mounted and coverslips. The images were obtained using a confocal microscope (Leica, Heidelberg, Germany).

For whole-mount analysis, the eyes were enucleated and fixed using 4% buffered neutral formalin fixatives (Biosharp, Beijing, China) at room temperature for 2 h. The connective tissue, muscle, and optic nerve were removed from the back of the eye, and the cornea and lens were removed to form an eyecup. The four radial incisions were made, and the retina was carefully dissected off the RPE/choroid under a dissecting microscope, and its connection to the optic nerve was severed. The RPE-choroid-sclera complexes, now separate samples, were further fixed in the round-bottom microcentrifuge tubes at room temperature for 1 h. The RPE eye-cups were flat-mounted and prepared for immunohistochemistry by blocking them with 10% normal goat serum in 0.3%. The tissues were incubated in triton X-100 in PBS for 1 h at room temperature. They were then incubated overnight at 4 °C with COX1 (Abcam #ab109025), COX2 (Cayman Chemical Company #160,107), CD206 (Abcam, #ab8918), CD80 (Abcam #ab86473), F40/80 (Abcam #ab6640), CD31 (Abcam #ab24590) and EP_1_R (Cayman Chemical Company #101,740). After washing the RPE flat mounts, they were incubated for 1 h with a secondary antibody (Thermo Fisher Scientific). The RPE flat mounts were then washed and counterstained with DAPI (Sigma, MO, USA) and examined using a confocal microscope (Leica, Heidelberg, Germany).

#### ELISA

Mouse PGE2 and IL-10 in the peripheral blood of mice was measured using commercial ELISA kits (Cayman Chemical Company #514,010; CUSABIO #CSB-E04594m) according to the manufacturer’s instructions according to the manufacturer’s instructions. The measure of each sample was repeated at least six times.

### Dual-luciferase reporter assay

The HEK293T cells were cultured in the RPMI medium 1640 basic DMEM (Gibco, Thermo Fisher) supplemented with 10% (v/v) fetal bovine serum at 37 °C. All the luciferase reporter plasmids were constructed by using a pGL3-Basic Vector. The HEK293T cells were separately transfected with pGL3-I1 (complete promoter IL-10 plasmid, 2000 bp), pGL3-I2 (truncated mutant IL-10 plasmid, 1500 bp), pGL3-I3 (truncated mutant IL-10 plasmid, 1000 bp), pGL3-I4 (truncated mutant IL-10 plasmid, 500 bp) and PGL3 basic (the negative control construct) for 48 h, and then supplemented with 5 μM PGE_2_. The relative luciferase activities were measured using the Dual-Luciferase® Reporter (DLR™) Assay System (Promega, #E1910) according to the protocol of the manufacturer.

### Electrophoretic mobility shift assay (EMSA)

The analysis of the NF-κB binding activity in the nuclear proteins was performed as described in our previous study [18]. The NF-κB binding activity was examined using a Light Shift Chemiluminescent EMSA kit (Thermo Fisher Scientific #20,148) according to the manufacturer’s instructions. Briefly, the nuclear proteins (5 μg) were isolated and specific unlabeled NF-κB competitors (50- and 100-fold molar excess) were used along with the binding reaction mixture for the competition assay. The biotin end-labelled DNA duplex of sequences containing the NF-κB binding site (5′-AGT TGA GGC GAC TTT CCC AGG C-3′, 3′-TCA ACT CCG CTG AAA GGG TCC G-5′) was incubated with the nuclear proteins at room temperature for 20 min. The reaction mixture was loaded onto 6% non-denaturing polyacrylamide gels and subsequently transferred to a nylon membrane (Hybond N^+^, Amersham Corp., Arlington Heights, IL). The membranes were exposed to ultraviolet light to cross-link proteins for 1 min and incubated with the conjugate/blocking buffer with the stabilized streptavidin horseradish peroxidase conjugate. The signal on the membranes was detected with the enhanced chemiluminescence system (West Pico kit, Pierce, Loughborough, UK). The membranes were then exposed to X-ray film for 2–6 min and the relative intensities were analysed using the Image J software (National Institutes of Health imaging software).

### Viability and proliferation (WST-1) assay

The cell proliferation reagent WST-1 (Beyotime Biotechnology, Shanghai, China) was used to assess the viability in 96-well culture cell plates as described previously [19]. Briefly, the treated cells (5 × 10^4^) were stained with WST at 37 °C for 2 h and quantified by measuring the absorbance at 450 nm and normalized with the control absorbance at 690 nm.

### Transwell assay

The cell migration assays were performed in 12-well hanging insert units (Millipore, Billerica, MA, USA). Before the experiment, 1 ml DMEM was added to the lower chamber of the transwell and incubated overnight. HRMECs (5 × 10^4^) were seeded to the upper chamber and 1 ml complete DMEM was added to the lower chamber of the transwell with pharmacological agents at the indicated time. After incubation at 37 °C for 12 h, the cells were fixed with 4% paraformaldehyde (wt/vol.) and then stained with 0.1% crystal violet for 30 min at room temperature. After the washing steps, the cells were removed using a moist cotton swab from the upper surface of the membrane. The cells that migrated to the lower surface of the membrane were solubilized with 300 μl of 10% acetic acid and observed under a fluorescence microscope (EVOS FL Auto Imaging System, Life Technologies).

### Wound scratch assay

The cells were seeded in 6-well plates. The wounding was performed by drawing a line with a pipette tip, and cells were washed twice with 1 × PBS. Next, the cells were treated with 25 or 50 ng/ml IL-10 for different times (0, 12, 24, 48 h). The gap size was observed under a microscope and assessed by the Image-Pro Plus software (Silver Spring, MD, USA).

### Western blot analysis

Western blotting and immunoprecipitation were conducted as previously described [20]. The cell and tissue proteins were lysed and isolated in the RIPA lysis buffer including protease inhibitor and phosphatase inhibitors for 30 min at 4 °C. The protein was obtained after centrifugation at 12000xg for 10 min, and the protein concentration was detected and quantified by the Pierce BCA Protein Assay Kit (Thermo Scientific, #23,225). An equal amount of protein was added to the 10% SDS-PAGE gel and transferred onto PVDF membranes. The membrane was incubated with the primary antibodies overnight at 4 °C. Then the membrane was washed three times with TBST for 5 min and incubated with the secondary antibodies at room temperature for 1 h. The primary antibodies are detailed in the Additional File 1: Table S1. The secondary antibodies included the HRP-labelled goat anti-rabbit IgG (ZSGB-BIO #ZB-2301, Beijing, China) and HRP-labelled goat anti-mouse IgG (ZSGB-BIO #ZB-2305, Beijing, China). The *β*-actin antibody (Sigma-Aldrich #A5316) and the Lamin B antibody (Abcam #ab16048) were used to confirm equal protein loading among the samples. The signals were detected with an enhanced chemiluminescence system (West Pico kit, Pierce, Loughborough, UK). The band density was analysed using the Image J software (National Institutes of Health imaging software).

### Quantitative real-time PCR

RNA was extracted and collected with RNA iso Plus (Takara, Cat#9109). The mRNA expression of mouse *Ep*_*1*_*r*, *Ep*_*2*_*r*, *Ep*_*3*_*r*, *Ep*_*4*_*r*, *Arg1*, *Mrc2*, *Mgl1*, *Ym1*, *Inos*, *Tnfa*, *Cd16*, *Cd32*, *Il-10* was determined by qRT-PCR. Gene expression was analyzed with the 2^−ΔΔCt^ method and normalized by an internal control, *Gapdh*. More details with primers are presented in Additional File 1: Table S1.

### Statistical analysis

The statistical analyses were analyzed with the GraphPad Prism-5 statistical software (Prism v5.0; GraphPad Software, La Jolla, CA, USA). All data are reported as mean ± SEM. We used Student’s t test to compare the mean of two groups. ANOVA with Tukey’s post hoc test was used to compare multiple groups. Results were considered statistically significant at *P* < 0.05.

## Results

### Macrophage activation is involved in laser-induced CNV in a mouse model of AMD

**Fig. 1:**
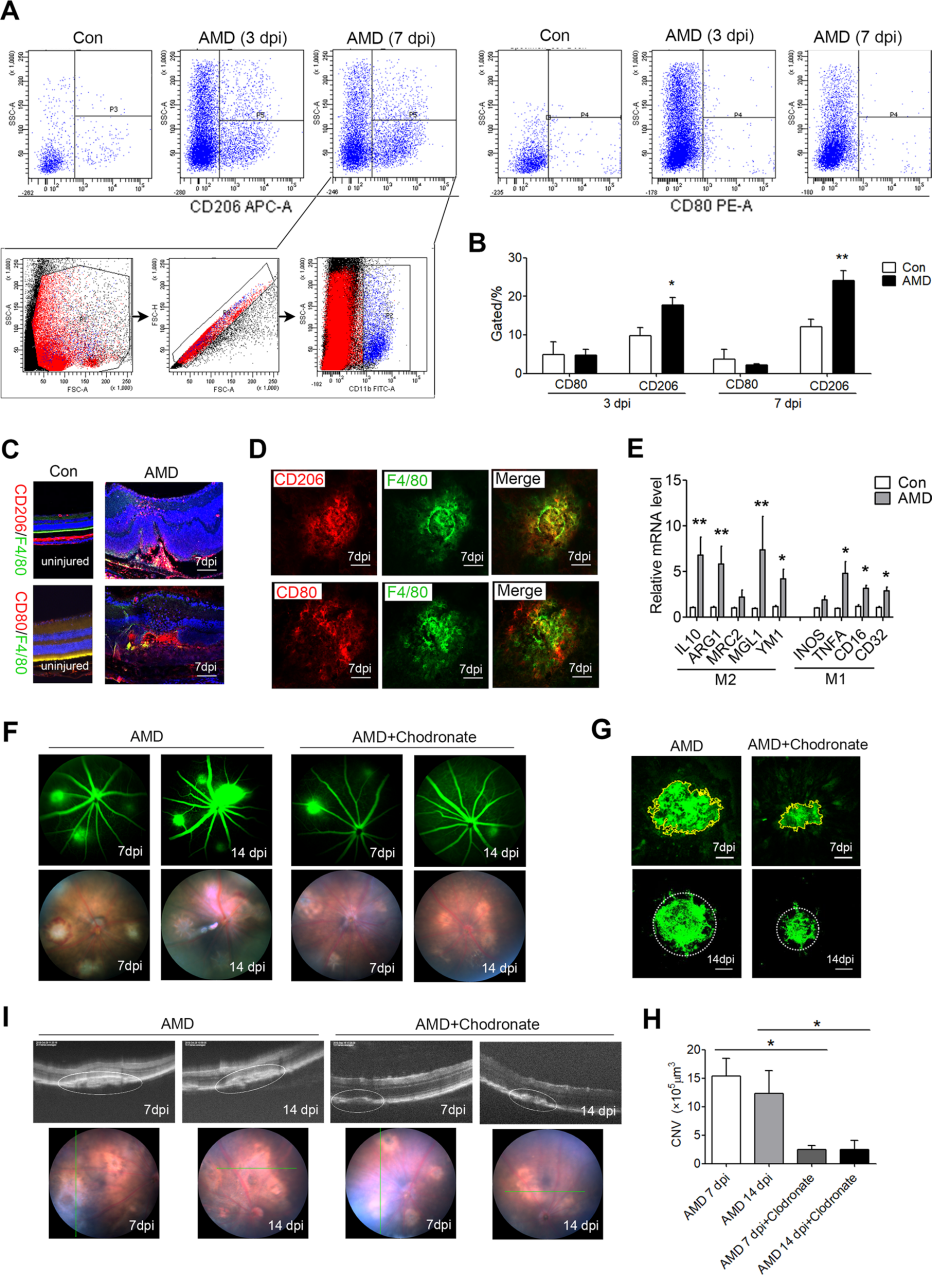
The activation of macrophages plays a key role in CNV development. **A, B** The proportion of CD206( +) or CD80( +) cells among CD11b-gated monocytes derived from the laser-induced mice at 3 dpi and 7 dpi was detected by flow cytometric analyses. **C, D** Immunofluorescence staining of the choroidal sections **C** and flat mounts **D** from the laser-induced mice (7 days post-injury [dpi]) with anti-F4/80 (green), anti-CD206 (red) and anti-CD80 (red) mAbs. **E** The mRNA levels of the M1 and M2-specific markers were measured by qRT-PCR at 7 dpi (*n* = 6). **F** The mice were treated with an intravitreal injection of 2 μl/eye of 90 μg clodronate liposomes. The representative vascular leakage image of FFA at 7 dpi and 14 dpi. **G** The immunofluorescence staining of the RPE-choroid complex mounts from mice at 7 dpi and 14 dpi with anti-IB4 mAbs were presented; scale bar: 100 μm. **H** Quantification of the CNV lesion area in the choroidal flat mounts (*n* = 6). **I** Representative fundus and OCT image of the eyes after the laser-induced CNV model (7 dpi and 14 dpi). Scale bar: 100 μm **C**, **D** and **G**. ^*^P < 0.05; ^**^P < 0.01

Recent studies have shown that macrophages are recruited to the periphery of the retinal laser photocoagulation induced wet AMD [21], and the activation of M1 and M2 macrophages was examined in the laser-induced CNV mice. Next, we collected monocytes in the eyes for flow cytometry 3 days and 7 days post-injury (3 dpi and 7 dpi). The proportions of CD206( +) cells among CD11b-gated monocytes derived from the laser-induced mice at 3 dpi and 7 dpi were significantly higher, when compared with the unlasered controls (Fig. 1A, B). In comparison, the percentage of CD80( +) CD11b( +) monocytes did not significantly change in the CNV mice (Fig. 1A, B). Consistently, the CD206^+^ & F4/80^+^ macrophages were found to infiltrate the periphery of the laser-injured area in the RPE-choroid in aged mice at 7dpi. At the same time, there was a slight increase in the CD80^+^ & F4/80^+^ macrophage levels in the laser-induced area (Fig. 1C, D). The expression levels of the mRNA for the M2-specific markers including *Il-10*, *arginase-1* (*Arg1*), *mannose receptor C type 2* (*Mrc2*), *Cd301b* (*Mgl2*), and *Chil3* (*Ym1*) or the M1 specific markers, *Inos* (*Nos2*), *Tnfα*, *Cd16*, and *Cd32* also confirmed the M2 and M1 activation at 7 dpi, respectively (Fig. 1E).

To determine whether macrophages activation contribute to the development of CNV, the macrophages were depleted by IVI of 2 μl/eye of 90 μg clodronate liposomes as previously described [21]. FFA was performed 7 days and 14 days after laser photocoagulation to observe vascular leakage and establishment of new vessels. There were several well-defined hyperfluorescent leaking spots representing dye leakage, while there were fewer leakage spots in those of the clodronate liposomes treatment group (Fig. 1F). Macrophage depletion reduced CNV volume in the clodronate liposomes-treated mice at 7 dpi and 14 dpi (Fig. 1G, H). Moreover, as shown by OCT, the CNV volume was also decreased in the clodronate liposomes-treated group compared to the vehicle group (Fig. 1I).

### COX2/PGE_2_ signalling is involved in the laser-induced CNV

**Fig. 2:**
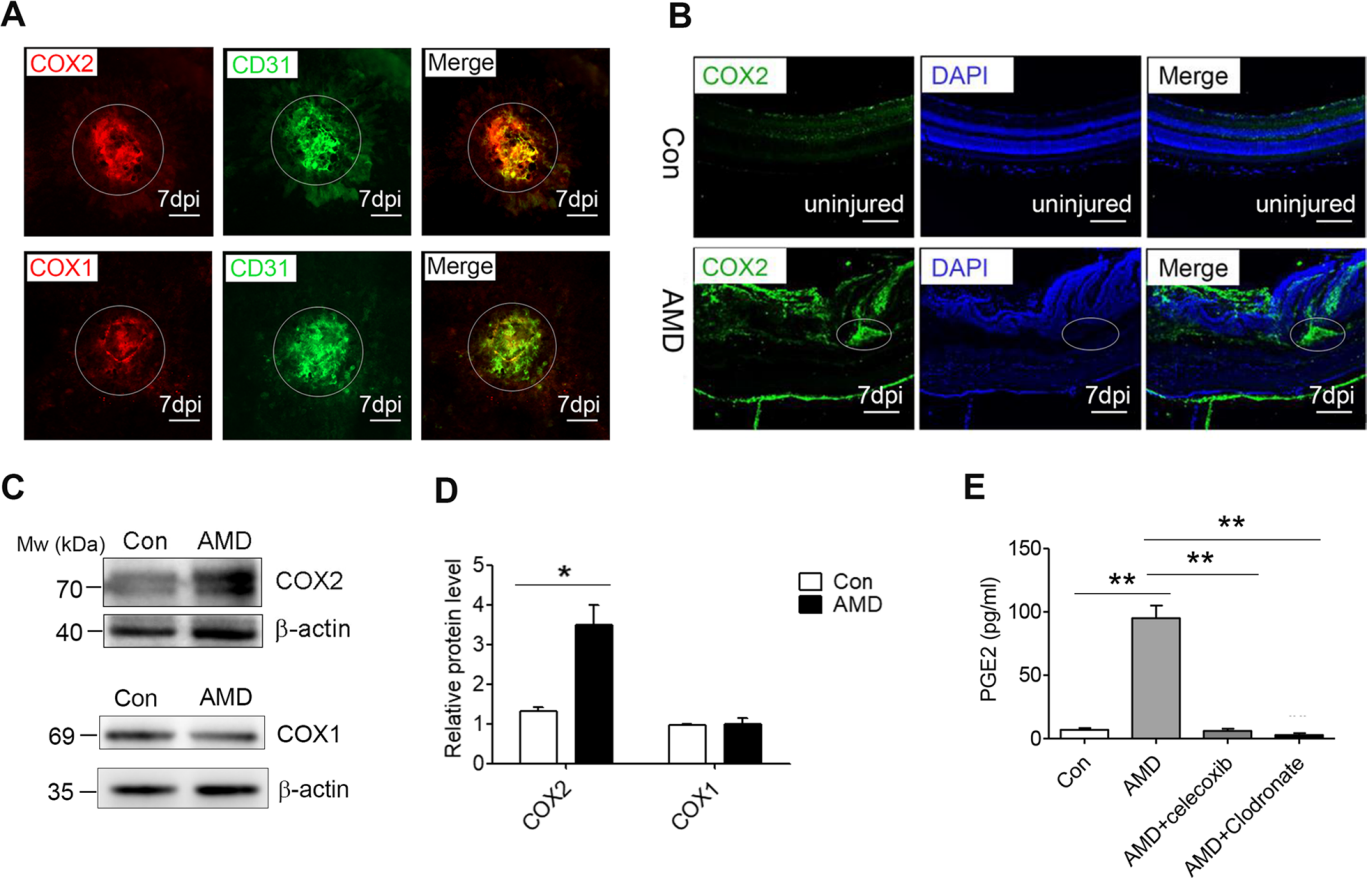
COX2 was highly expressed in the laser-injured RPE-choroid complex. **A**, **B** Immunofluorescence staining of the choroidal flat mounts **A** and sections **B** from mice (7 dpi) with anti-CD31, anti-COX2, and anti-COX1 mAbs; scale bar: 100 μm. **C, D** The COX2 and COX1 protein at 7 dpi was measured by western blotting using *β*-actin as an internal control. The representative blots are shown, with quantification (*n* = 6). **E** Quantitative analysis of PGE_2_ levels (*n* = 6). The level of PGE_2_ in the peripheral blood of mice (7 dpi) was measured by ELISA after the laser-induced CNV model. ^*^P < 0.05; ^**^P < 0.01

We evaluated the effect of prostanoid-associated signalling in the progression of laser-induced CNV. COX2 was dramatically increased in the periphery of the laser-injured area at 7 dpi in the RPE-choroid in the aged mice (Fig. 2A, B). COX1 showed no significant change in the flat-mounted RPE-choroid at 7 dpi (Fig. 2A). Moreover, as shown by western blot analysis, the amount of COX2 protein at 7 dpi was higher in the laser-treated eyes, while no significant increase was noted in COX1 expression (Fig. 2C, D). Consistent with the COX2 inhibitor, clodronate liposomes also significantly decreased the PGE_2_ production at 7 dpi in the vitreous fluid of the AMD mice (Fig. 2E). These data suggest that COX2/PGE_2_ signalling is associated with the progression of laser-induced CNV.

### Celecoxib treatment ameliorates the progression of CNV in a mouse model of AMD

**Fig. 3:**
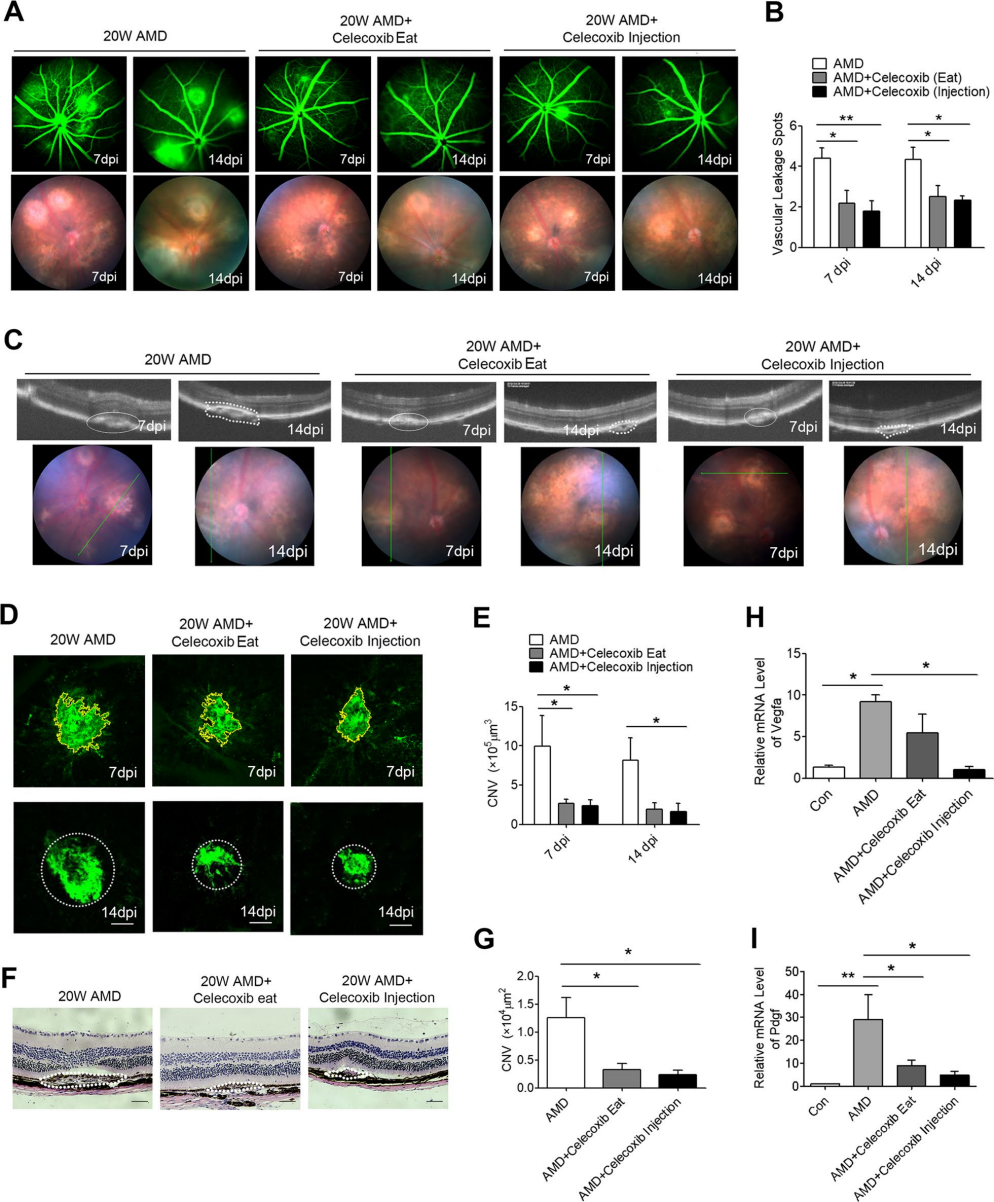
Celecoxib attenuates CNV in the mouse model of AMD. **A** Representative images of the vascular leakage at 7 dpi and 14 dpi are displayed. **B** Quantification of vascular leakage spots (*n* = 6). **C** The cross-sectional OCT and fundus images at 7 dpi and 14 dpi. **D** Immunofluorescence staining of the flat mounts from the mice at 7 dpi and 14 dpi with anti-IB4 mAbs. **E** Quantification of the CNV lesion area in the choroidal flat mounts (*n* = 6). **F, G** H&E staining of the paraffin sections of the choroidal Sects. (14 dpi) with quantification (*n* = 6). **H, I** The mRNA levels of Vegfa and Pdgf were measured in the RPE-choroid complex at 7 dpi by qRT-PCR (*n* = 6). Scale bar: 100 μm **D** and **F**. *P < 0.05; ^**^P < 0.01

Celecoxib is a highly selective COX2 inhibitor with anti-inflammatory and analgesic properties [22]. Rhodopsin kinase (GRK1) plays an important role in phototransduction and its level in the retina provides an assessment of rod photoreceptor cell survival and function. GRK1 level in the RPE-choroid was used to evaluate the cytotoxicity of celecoxib working solutions. The 2.5, 5 or 10 mM celecoxib treatment had no cytotoxicity to RPE-choroid in vivo. Although 10 mM celecoxib treatment slightly increased the GRK1 expression (Additional file 1: Figure S1A and B). There was also no significant cytotoxicity of celecoxib working solutions at the concentration of 2.5–10 mM shown by the funds and OCT images (Additional file 1: Figure S1C). The 2.5, 5, 10 μM celecoxib treatment also had no cytotoxicity to BMDMs (Additional file 1: Figure S1D). In the laser-injured mice, the effects of COX2 on retinal vascular leakage were evaluated. As shown by FFA, there were several intense hyperfluorescent spots at 7 dpi and 14 dpi in the laser-injured mice, while there were fewer leakage spots in the celecoxib intravitreal injected or celecoxib fed mice (Fig. 3A, B).

The CNV volume shown by OCT and iB_4_ staining was decreased in the celecoxib-treated groups, compared to the vehicle control group (Fig. 3C–E). Histological analysis revealed that the laser-induced AMD mice developed robust neovascularization in the retinal and choroidal tissues, which was partially restored in the celecoxib group (AMD group, 1.25 × 10^4^ μm^2^; celecoxib feeding group, 0.33 × 10^4^ μm^2^; celecoxib injection group, 0.23 × 10^4^ μm^2^) (Fig. 3F, G). Moreover, the expression of profibrotic factors Vegfa and Pdgf dramatically increased in the RPE-choroid of the aged mice at 7 dpi, and decreased in the celecoxib-treated mice (Fig. 3H, I).

### Celecoxib treatment ameliorates the activation of macrophages in the laser injured mice

**Fig. 4:**
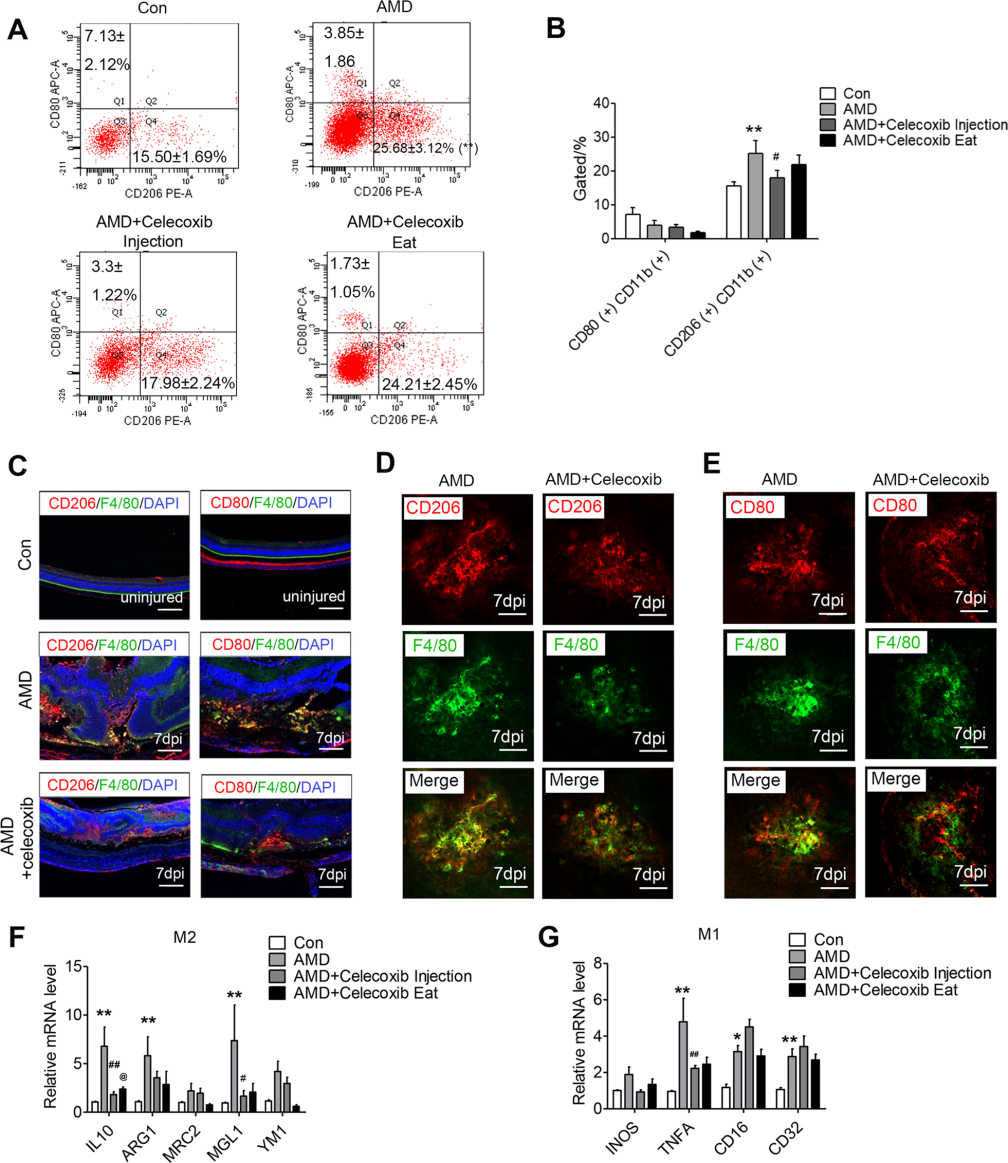
Celecoxib inhibits the macrophages activation in the CNV lesions. **A** The proportion of CD206 + or CD80 + cells among CD11b-gated monocytes derived from the laser-induced mice at 7 dpi was detected by flow cytometric analyses. **B** Column diagram is the percentages of the populations of CD11b-gated CD206 + or CD80 + in flow cytometric analyses (*n* = 6). **C–E** Immunofluorescence staining of choroidal sections and flat mounts from mice at 7 dpi with anti-CD206, anti-CD80 and F4/80 mAbs; scale bar: 100 μm. **F, G** The mRNA levels of the M1 and M2-specific markers were measured at 7 dpi by qRT-PCR in the RPE-choroid complex (*n* = 6). ^*^P < 0.05, ^**^P < 0.01 vs. Con; and ^#^*P* < 0.05, ^##^*P* < 0.01 vs. AMD; and ^@^*P* < 0.05 vs. AMD

We further collected monocytes in the eyes for flow cytometry at 7dpi, and the laser-induced increased percentage of CD206( +) CD11b( +) was potently inhibited by the celecoxib intravitreal injected (Fig. 4A, B). In comparison, the percentage of CD80( +) CD11b( +) monocytes did not significantly change in the CNV mice (Fig. 4A, B). We observed that the CD206^+^F4/80^+^ macrophages infiltrated the periphery of the laser-injured area at 7 dpi in the RPE-choroid. The IVI of celecoxib significantly reduced the CD206^+^F4/80^+^ macrophages in the RPE-choroid compared to that of the vehicle control. However, the CD80 + F4/80 + macrophages infiltrated the periphery of the laser-injured area at 7 dpi in the celecoxib-treated AMD mice showing a slight change vs vehicle control-treated AMD mice (Fig. 4C–E). The activation of macrophages was confirmed by examining the expression levels of mRNA at 7 dpi for the M2-specific markers or M1 specific markers in BMDMs of the laser-induced eyes (Fig. 4F, G).

### PGE_2_/EP_1_R/PKC signalling is involved in the activation of M2 macrophages

**Fig. 5:**
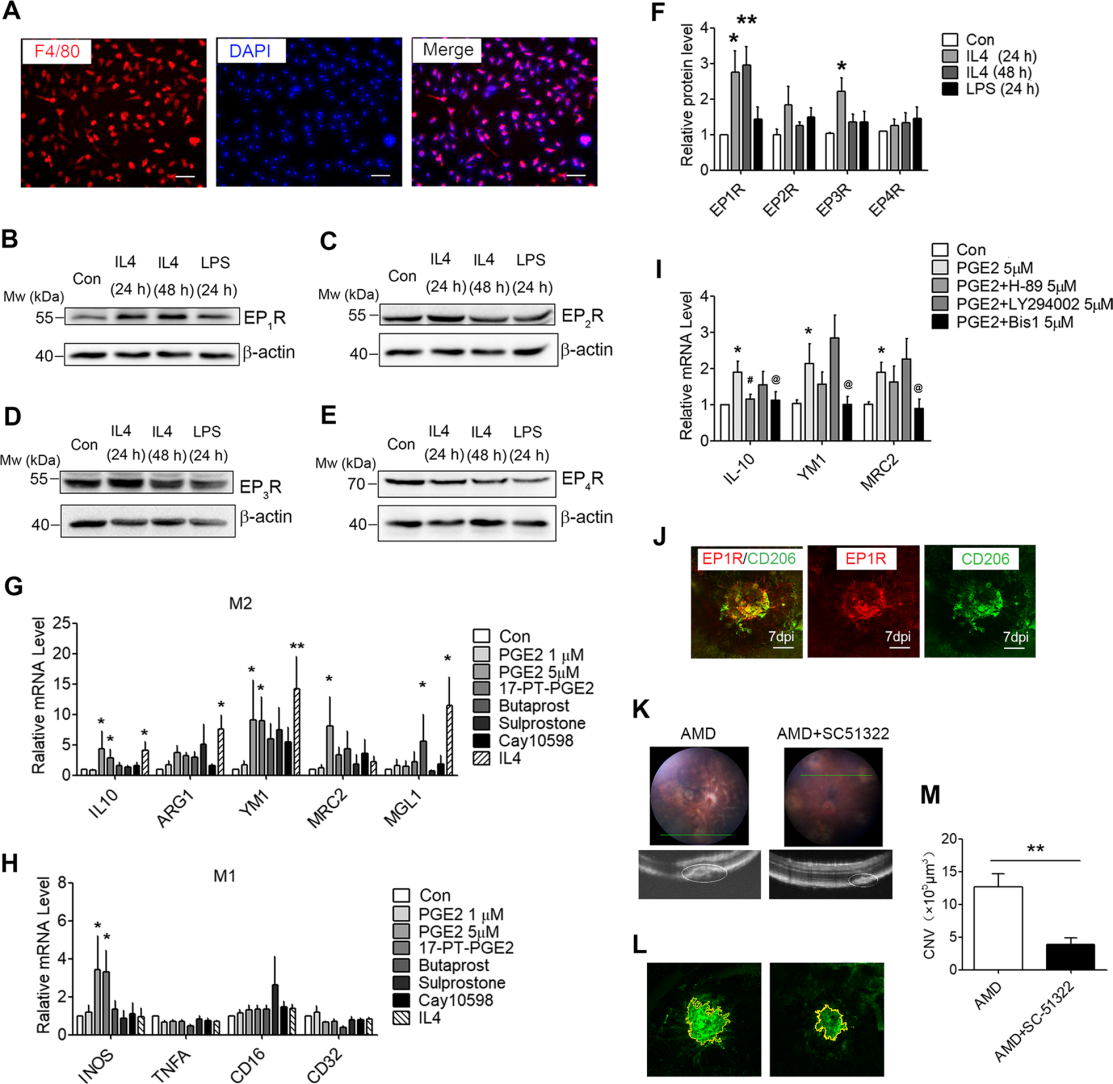
EP_1_R/PKC signalling is involved in macrophages activation **A** Immunofluorescence staining of BMDMs with F4/80 (red) and nuclei (blue) is presented; scale bar: 50 μm. **B–F** The protein expression levels of EP_1-4_R measured by western blot analysis using *β*-actin as the internal control. Representative blots are shown, with quantification (*n* = 3 per group). **G, H** The mRNA levels of the M1 and M2-specific markers were measured by qRT-PCR in the BMDMs treated with 1 μM PGE_2_, 5 μM PGE_2_, 10 μM 17-PT-PGE_2_, 10 μM Butaprost, 10 μM Sulprostone, 10 μM Cay10598, and 20 ng/mL IL-4, respectively. **I** The quantitative PCR analysis of IL-10, Ym1, and MRC2 expression in the BMDMs pretreated with 5 μM H89, 5 μM LY294002, and 15 μM Bis 1 for 30 min respectively before being supplemented with 5 μM PGE_2_ for 24 h (*n* = 3). **J** Immunofluorescence staining of choroidal flat mounts from the mice with anti-EP_1_R and anti-CD206 mAbs; scale bar: 100 μm. **K** Representative fundus and OCT image of the eyes after the laser-induced CNV model. **L** Immunofluorescence staining of the flat mounts from the mice with anti-IB4 mAbs. **M** Quantification of the CNV lesion area in the choroidal flat mounts (*n* = 6); ^**^P < 0.01 vs. Con; and ^#^*P* < 0.05 vs. PGE2 5 μM; and ^@^*P* < 0.05 vs. PGE2 5 μM

LPS or IFN-*γ* induce a classical activation of macrophages (M1), whereas IL-4 or IL-13 induce an alternative activation program in the macrophages (M2) [8]. We further examined whether EP_1–4_Rs are involved in CNV formation in the macrophages in vitro. We isolated the mouse bone marrow cells by adding M-CSF to induce differentiation into BMDMs after 7 days. Immunofluorescence staining revealed that the positive rate of the F4/80-labeled BMDMs reached 95% (Fig. 5A). The M2 polarization of BMDMs significantly induced EP_1_R protein expression at various time points (24 h and 48 h) in response to the IL-4-stimuli, while the M2 polarization also showed a slight increase in the EP_3_R expression at 48 h (Fig. 5B–F). The BMDMs were treated with PGE_2_ and an EP_1_R agonist (17-PT-PGE_2_), EP_2_R agonist (Butaprost), EP_3_R agonist (Sulprostone), and EP_4_R agonist (Cay10598) to investigate whether EP_1-4_Rs are the potential regulators of M2 polarization. IL-4 was used as a positive control. Indeed, 5 μM PGE_2_ or EP_1_R agonist could induce the activation of M2 macrophages at 24 h (Fig. 5G, H). As PKA, PKB/Akt, and PKC are the critical intracellular kinases involved in PGE_2,_ activating the EP_1-4_Rs signalling, the effects of a PKA inhibitor (H89), PKB inhibitor (LY294002), and PKC inhibitor (Bis1) were examined in the PGE_2_-induced macrophage activation. The Bis1 pre-treatment for 30 min significantly decreased the PGE_2_-induced M2-specific markers expression at 24 h, while there was no significant difference in the H89 or LY294002-treated groups (Fig. 5I). Meanwhile, the EP_1_R was co-expressed with the CD206 at 7 dpi in the RPE-choroid mounts of the aged mice (Fig. 5J). The CNV volume shown by OCT was decreased in the EP1R antagonist (SC51322)-treated groups, compared to the vehicle control group (Fig. 5K). IB4 staining was decreased in the SC51322-treated groups, compared to the vehicle control group (average area in the AMD mice, 12.67 × 10^5^ μm^3^; SC51322 injection mice, 3.85 × 10^5^ μm^3^) (Fig. 5L, M). Taken together, these data suggest that PGE_2_/EP_1_R/PKC signalling mediates the activation of the M2 macrophages in the laser-induced CNV.

### H_2_O_2_ induces the polarization of M2 macrophages of BMDMs

**Fig. 6:**
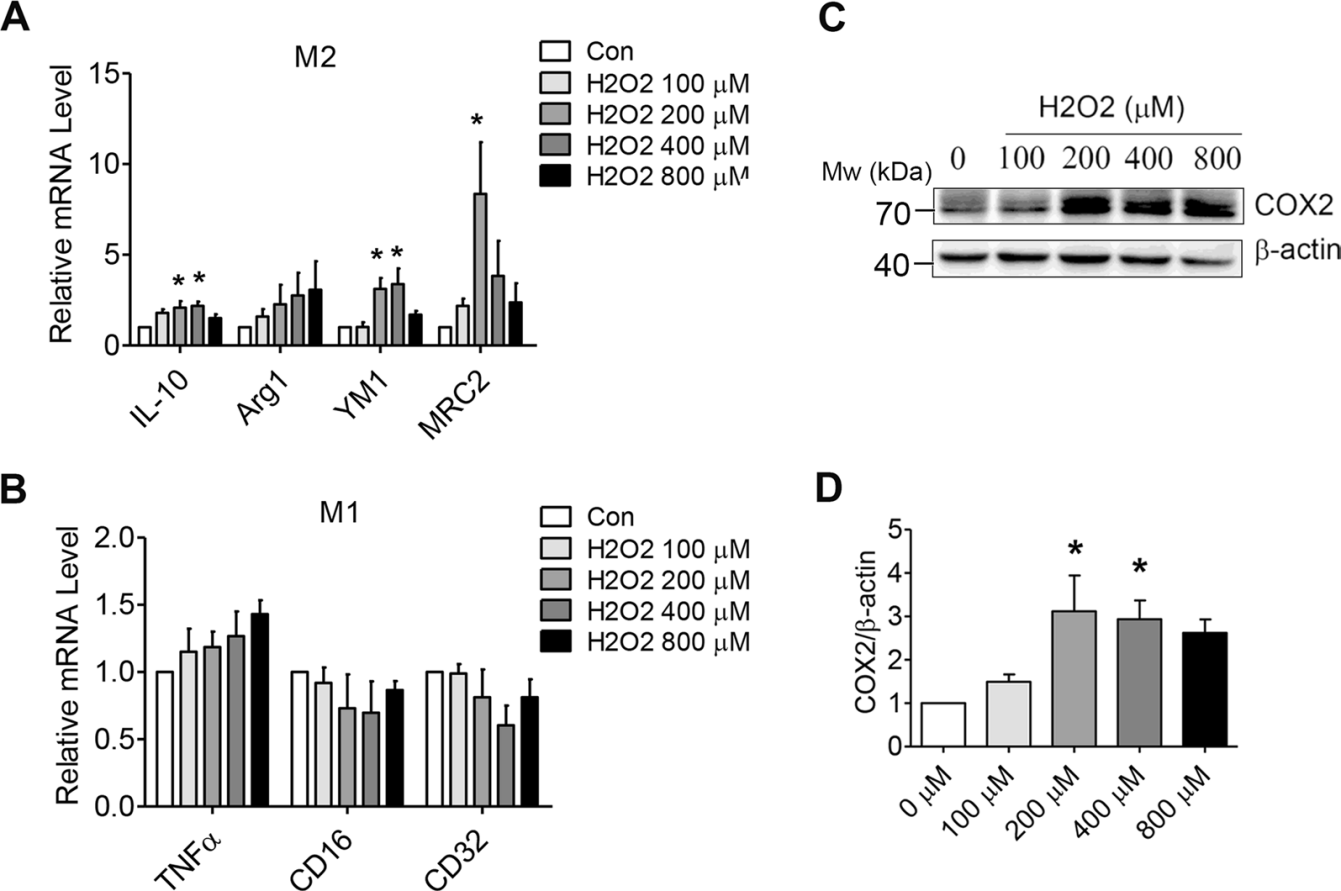
H_2_O_2_ induces M2 polarization in BMDMs. **A, B** The mRNA levels of the M1 and M2-specific markers were measured by qRT-PCR in the BMDMs (*n* = 3). **C, D** COX2 protein was measured by western blotting using *β*-actin as an internal control. Representative blots are shown, with quantification (*n* = 3). ^**^P < 0.01, ^***^P < 0.001

The pathogenesis of wet AMD involves a variety of cellular processes, including oxidative stress, abnormal cell metabolism, and impaired cell function [2]. To test the consequences of increased oxidative stress on macrophage activation of BMDMs, the cells were incubated in different concentrations of H_2_O_2_ (0, 100, 200, 400, and 800). After 8 h, there was a significant increase in the expression of the M2-specific marker, while there was no significant difference in M1-specific markers expression between the groups (Fig. 6A, B). H_2_O_2_ treatment for 24 h resulted in a significant increase in the COX2 expression compared to the control medium (Fig. 6C, D).

### PGE_2_ exacerbates the IL-10 secretion level by increasing the NF-*κ*B binding activity

**Fig. 7:**
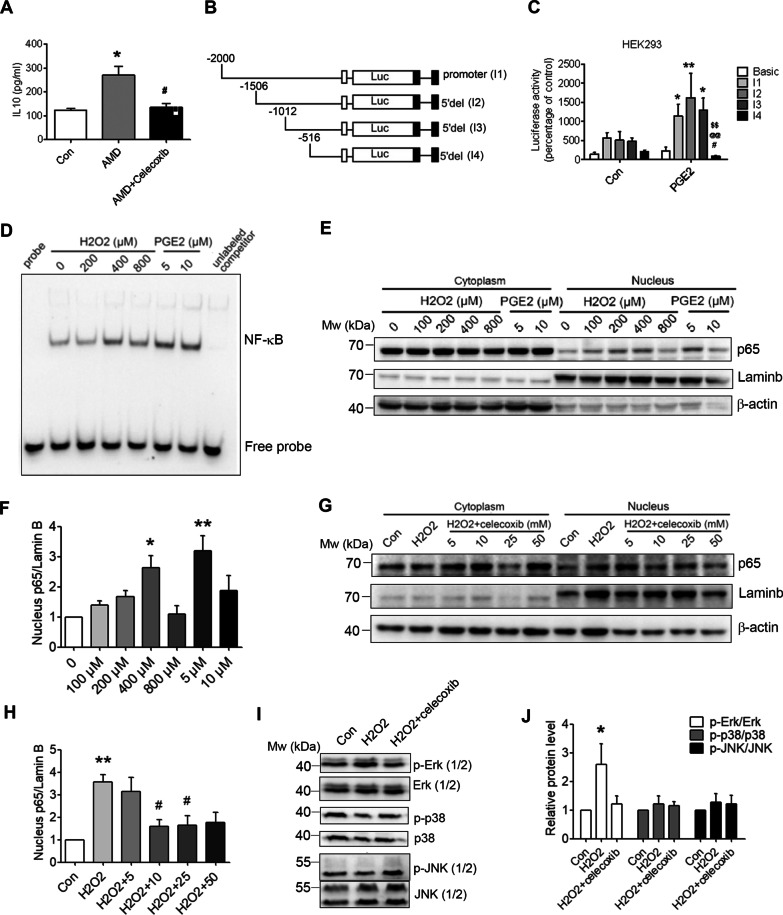
PGE_2_ regulates IL-10 secretion level by increasing the NF-*κ*B binding activity. **A** Plasma IL-10 was determined by ELISA in mice (*n* = 6); ^*^P < 0.05 versus Con group; ^#^*P* < 0.05 versus AMD group. **B, C** Effects of PGE_2_ on the luciferase activity in the HEK293 cell line transiently transfected with PGL3 basic, PGL3 I1, PGL3 I2, PGL3 I3, and PGL3 I4 (*n* = 4); ^*^P < 0.05 versus Basic group; ^**^P < 0.01 versus Basic group; ^#^*P* < 0.05 versus I1 group; ^@@^*P* < 0.01 versus I2 group; ^$$^*P* < 0.01 versus I3 group. **D** Nuclear and cytoplasmic proteins isolated from BMDMs. The NF-*κ*B binding activity was detected by EMSA (*n* = 3). **E–H** P65-NF-κB protein was measured by western blotting using β-actin as the cytoplasmic control and Lamin b as the nuclear control. Representative blots are shown, with quantification (*n* = 3); ^*^P < 0.05, ^**^P < 0.01 versus Con group; ^#^*P* < 0.05 versus H_2_O_2_ group. **I, J** The levels of phospho-ERK/ERK, phospho-JNK/JNK, and phospho-p38/p38 were determined by western blotting. Quantification of the western blot band intensity is shown in the respective right panels (*n* = 3); ^*^P < 0.05

As an M2-specific marker, IL-10 promotes alternative macrophage activation and pathological neovascularization [10]. In our study, we also found that IL-10 was significantly increased at 7 dpi in the laser-induced eyes, which was reversed by the pre-treatment with celecoxib (Fig. 7A). We further identified the region of the IL-10 promoter that was important for mediating the inductive effects of PGE_2_. The transient transfections were performed with a series of human IL-10 promoter-deletion constructs (Fig. 7B). Treatment of the HEK293 cells with PGE_2_ led to a several-fold increase in the IL-10 promoter activity when the -2000 bp deletion construct (I1) was used (Fig. 7C). The magnitude of PGE_2_-mediated induction of the IL-10 promoter activity remained essentially constant until the − 516 bp deletion construct (I3) was used. The − 516 bp IL-10 promoter construct (I4) was not stimulated by PGE_2_ (Fig. 7C). This result implied that one or more promoter elements located between − 516 bp and − 1012 bp is necessary for the PGE_2_-mediated induction of the IL-10 promoter activity. NF-κB sites are found within this region of the IL-10 promoter [23]. Recent studies have reported that NF-κB plays important role in the early stages of angiogenesis and CNV [24]. NF-κB is also known to regulate several genes involved in angiogenesis (VEGF, intercellular adhesion molecule 1 [ICAM1] and COX2) [25, 26]. As oxidative stress serves a vital function in the pathogenesis of AMD, in our study, the BMDMs were also subjected to H_2_O_2_ treatment to induce oxidative damage. The results revealed that 400 μM H_2_O_2_ or 5 μM PGE_2_ dramatically induced the DNA binding activity of NF-κB (Fig. 7D). Meanwhile, treatment of the BMDMs with H_2_O_2_ or PGE_2_ could increase the NF-κB translocation from the cytoplasm to the nucleus (Fig. 7E, F). Besides, 10 μM celecoxib treatment dramatically prevented the H_2_O_2_-induced p65 expression in the nucleus (Fig. 7G, H).

MAPKs, including Erk (1/2), p38 and JNK (1/2), mediated the activation of NF-κB, which have been demonstrated to play an important role in oxidative stress [27]. As shown in Fig. 7, H2O2 induced a significant increase in the phosphorylation of Erk (1/2) compared to the control group, which was prevented in the celecoxib-treated group. However, there was no significant difference in the level of phospho-p38 or phospho-JNK (1/2) among the groups (Fig. 7I, J).

### Role of IL-10 in the proliferation and migration of the human choroidal microvascular endothelial cells (HCECs)

**Fig. 8:**
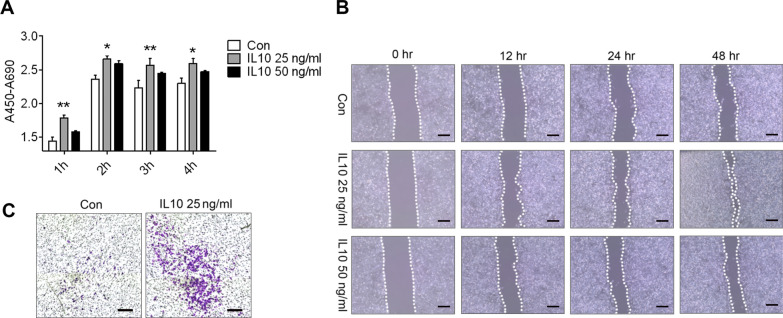
IL-10 promotes cellular proliferation and migration in hRMECs. **A** Statistical analysis of the viability of hRMECs by the CCK8 assay (*n* = 4). **B** Cell migration was assessed using a wound-healing assay. Images were taken 0, 12, 24, and 48 h after IL-10 treatment. Migration was estimated by measuring the cell numbers within the wounded region; scale bar, 50 μm. **C** Migration of hRMECs in the transwell assays was determined; scale bar, 25 μm. ^*^P < 0.05; ^**^P < 0.01

The role of IL-10 in HCECs proliferation and migration were examined using the WST-1 and transwell migration assays, as well as by observing the distance in wound healing. The proliferation and migration were dramatically increased in the 10 ng/ml or 25 ng/ml IL-10-treated HCECs (Fig. 8A–C).

## Discussion

The present study demonstrated the important role of the PGE_2_/EP_1_R signalling pathway in the macrophages activation in the pathogenesis of CNV in wet AMD (Graphical abstract).

PGE_2_ is an important factor for cell growth and regulation, which is a metabolite of the arachidonic acid COX, and their role has been reported in many disorders, including tumour metastasis, rheumatoid arthritis, asthma and inflammatory diseases [28–30]. Increasing evidence has proved that PGE_2_ is involved in the retinopathy of prematurity (ROP), diabetic retinopathy, pterygium and other ocular neovascular diseases [18]. We found that the PGE_2_ expression increased in the eyes after induction of the laser injury. COX2 is an enzyme inducible by pathologic stimuli such as lipopolysaccharides, IL-1*β*, TNF-*α*, and NF-*κ*B [31]. The expression of COX2 in human choroidal neovascular membranes was related to the AMD pathology by increasing the secretion of the VEGF and TGF-β [32]. We found that laser photocoagulation could induce a significant increase in the volume of CNV in the vehicle control-treated mice, but not in the celecoxib-treated mice. COX2 and EP_1_R, which were expressed in the periphery of the laser-injured area in the RPE-choroid after laser injury in the aged mice were responsible for CNV.

A previous study reported that the M2 macrophages recruited to the injured eyes highly contribute to the pathogenesis of CNV in wet AMD [21]; here, we also demonstrated that the ocular-infiltrating M2 macrophages contributed to the CNV development involving the PGE_2_/EP_1_R signalling pathway. Indeed, the laser-induced injury increased the amount of the M2 macrophages to the injured mouse retina, however, the inhibition of macrophages activation remarkedly inhibited the laser-induced CNV progression. We also found that PGE_2_ or 17-PT-PGE_2_ or H_2_O_2_ could mainly induce the M2 polarization activation in the primary mouse macrophages. Blockade of the PGE_2_ signalling strongly inhibited the macrophages activation.

Recent studies have shown that the expression of IL-10 and M2 markers (e.g., CD163 and Arginase-1), but not M1 markers (e.g., IL-6 and TNF-α), are increased in the senescent macrophages, thereby promoting the CNV development [10]. Two main subtypes of M2 macrophages have been implicated in wound healing, namely those stimulated (at least in vitro) by interleukin-4 (IL-4) (called M2a) and those stimulated by IL-10 (called M2c) [9]. Previously, studies have shown that the IL-10-stimulated M2c macrophages promoted more angiogenesis in vitro and in vivo compared to the M1 and M2a phenotypes [10]. We found that IL-10 was significantly increased in the laser-induced AMD mice, which was blocked by the celecoxib treatment. In vitro*,* the increased production of IL-10 in H_2_O_2_ or the PGE_2_-treated BMDMs was determined. Meanwhile, IL-10 could induce the proliferation and migration of the HCECs. NF-*κ*B is a key mediator that regulates the inflammatory response. It is also an upstream regulator of COX2. Inhibition of NF-κB signalling can reduce the inflammatory expressions and angiogenic factors in the RPE cells. Our results revealed that PGE_2_ and H_2_O_2_ significantly induced DNA binding activity of NF-κB and phosphorylation of Erk (1/2), which was reversed by the celecoxib treatment.

## Conclusions

To summarize, the present study provides strong evidence that disruption of the PGE_2_/EP_1_R signalling pathway contributes to the attenuation of the CNV pathogenesis. The underlying mechanism is manifold. Treatment of PGE_2_ resulted in the IL-10 production and macrophages activation in the laser-induced CNV model (Graphical abstract). These results indicate that PGE_2_/EP_1_R mediated macrophages activation by promoting the activation of the Erk (1/2)/NF-κB signalling network and that this network might represent a potential therapeutic target for postnatal retinal angiogenesis.

## Supplementary Information

**Additional file 1: Fig. S1: **The cytotoxicity of intravitreal injection of celecoxib. **Table S1.** Key resources table

## Data Availability

The data that support the fundings of this study are available from the corresponding author upon reasonable request. All data generated or analysed during this study are included in this published article.
