# Supplementary material for: PGE2 promotes macrophage recruitment and neovascularization in murine wet-type AMD models
Source: Cell Commun Signal. 2022 Oct 13;20:155. doi: 10.1186/s12964-022-00973-6 (PMC9558420; doi:10.1186/s12964-022-00973-6)
Supplement: Supplementary file 2 — Additional file 1: Fig. S1: The cytotoxicity of intravitreal injection of celecoxib. Table S1. Key resources table [file 12964_2022_973_MOESM2_ESM.docx]

# PGE2 promotes macrophage recruitment and neovascularization in murine wet-type AMD models

Pengfei Zhan^1^ & Yuqing Cui^1^ & Yujuan Cao^1, 2, 3^ & Xun Bao^1^ & Meili Wu^2^ & Qian Yang^1^ & Jiahui Yang^1^ & Haohan Zheng^1^ & Jian Zou^2^ & Tianhua Xie^1^ & Jiping Cai^1^ & Yong Yao*^1, 3^ & Xiaolu Wang*^2^

^1^ Department of Ophthalmology, The Affiliated Wuxi People's Hospital of Nanjing Medical University, 299 Qingyang Road, Wuxi, Jiangsu 214023, People’s Republic of China

^2^ Center of Clinical Research, The Affiliated Wuxi People's Hospital of Nanjing Medical University, 299 Qingyang Road, Wuxi, Jiangsu 214023, People’s Republic of China

^3^ Department of Ophthalmology, The Affiliated Wuxi No.2 People's Hospital of Nanjing Medical University, Wuxi, Jiangsu 214023, People’s Republic of China

**Correspondence**

**Yong Yao**, Department of Ophthalmology, The Affiliated Wuxi People's Hospital of Nanjing Medical University, 299 Qingyang Road, Wuxi, Jiangsu 214023, People’s Republic of China

E-mail: [yongyao@njmu.edu.cn](https://d.docs.live.net/ff3861900cb5b517/杂项%20writing/yongyao@njmu.edu.cn)

ORCID: 0000-0002-5506-7101

**Xiaolu Wang**, Center of Clinical Research，The Affiliated Wuxi People's Hospital of Nanjing Medical University, 299 Qingyang Road, Wuxi, Jiangsu 214023, People’s Republic of China

E-mail: [xlwang@njmu.edu.cn](mailto:xlwang@njmu.edu.cn)

ORCID: 0000-0001-8089-6473

***Additional File 1***


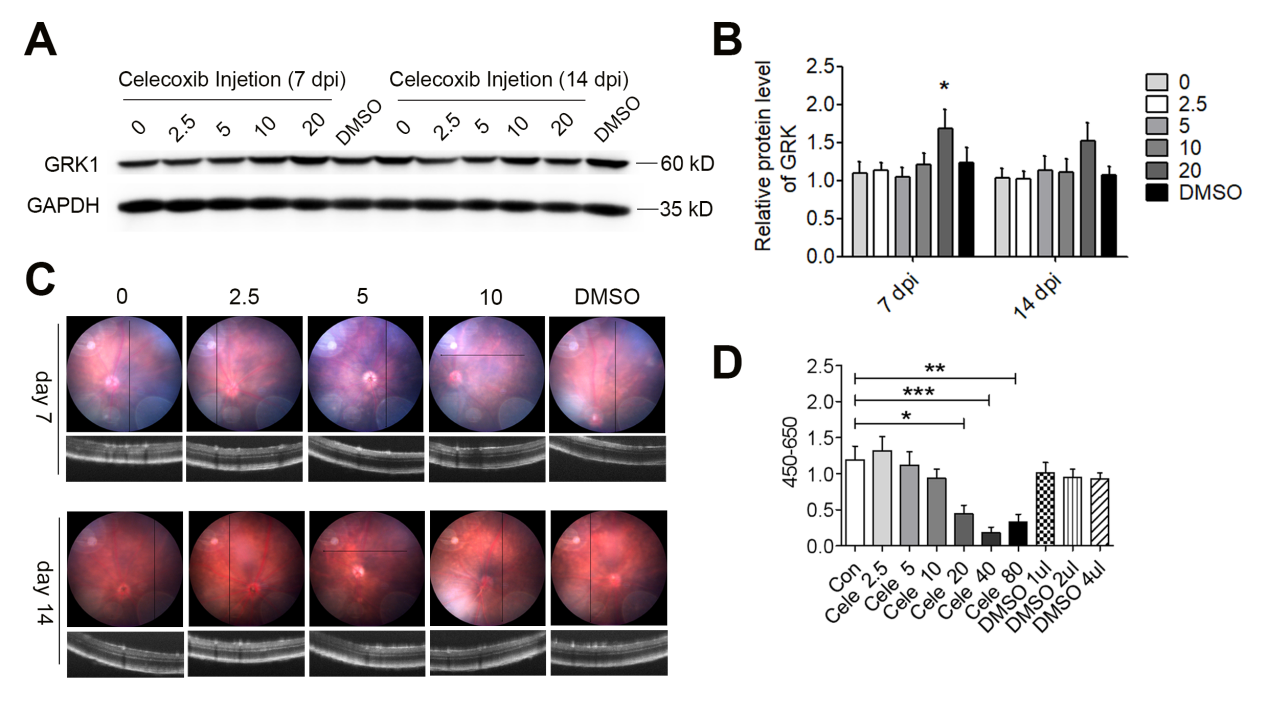


**Figure S1: The cytotoxicity of intravitreal injection of celecoxib.** Mice were administrated intravitreal injections of 2 μl saline, celecoxib diluents (2.5, 5, 10, 20 mM), and DMSO diluents (25%). (**A, B**) GRK protein was measured by western blotting using GAPDH as an internal control. Representative blots are shown, with quantification (n=6). (**C**) Representative funds and OCT images of the eyes at day 7 and day 14 post injection. (**D**) Cell viability was assessed using the cell proliferation reagent WST (n=4).

***Table S1. Key resources table***

| **Reagent type (species) or resource** | **Designation** | **Source or reference** | **Identifiers** | **Additional information** |
| --- | --- | --- | --- | --- |
| Antibody | Anti-COX1 (rabbit monoclonal) | abcam | Cat#ab109025  RRID: AB_10865291 | IF (1:400) |
| Antibody | Anti-CD80 (mouse monoclonal) | abcam | Cat#ab86473  RRID: AB_2076149 | IF (1:400) |
| Antibody | Anti-F4/80 (rat monoclonal) | abcam | Cat#ab6640  RRID: AB_1140040 | IF (1:200) |
| Antibody | Anti-EP4R (rabbit polyclonal) | abcam | Cat#ab45295 RRID: AB_882214 | WB (1:1000) |
| Antibody | Anti-CD206 (mouse monoclonal) | abcam | Cat#ab8918  RRID: AB_306860 | IF (1/50) |
| Antibody | Anti-CD31 (mouse monoclonal) | abcam | Cat#ab24590  RRID: AB_448167 | IF (1:1000) |
| Antibody | Anti-Laminb (rabbit polyclonal) | abcam | Cat#ab16048  RRID: AB_10107828 | WB (1:1000) |
| Antibody | Anti-COX2 (rabbit polyclonal) | Cayman Chemical Company | Cat#160107  RRID: AB_10078833 | WB (1:200) IF (1:200) |
| Antibody | Anti-EP1R (rabbit polyclonal) | Cayman Chemical Company | Cat#101740  RRID: AB_10079426 | WB (1:200) IF (1:200) |
| Antibody | Anti-EP2R (rabbit polyclonal) | Cayman Chemical Company | Cat#101750  RRID: AB_10078697 | WB (1:200) IF (1:100) |
| Antibody | Anti-EP3R (rabbit polyclonal) | Cayman Chemical Company | Cat#101760  RRID: AB_10077931 | WB (1:200) IF (1:100) |
| Antibody | Anti-p-p65 (rabbit monoclonal) | Cell Signaling | Cat#3033S  RRID: AB_331284 | WB (1:1000) |
| Antibody | Anti-p65 (rabbit monoclonal) | Cell Signaling | Cat#8242S  RRID: AB_10859369 | WB (1:1000) |
| Antibody | Anti-p-Erk (1/2) (rabbit monoclonal) | Cell Signaling | Cat#4370S RRID: AB_2315112 | WB (1:1000) |
| Antibody | Anti-Erk (1/2) (rabbit monoclonal) | Cell Signaling | Cat#4695S  RRID: AB_390779 | WB (1:1000) |
| Antibody | Anti-p-p38 (rabbit monoclonal) | Cell Signaling | Cat#4511S  RRID: AB_2139682 | WB (1:1000) |
| Antibody | Anti-p38 (rabbit monoclonal) | Cell Signaling | Cat#8690S  RRID: AB_10999090 | WB (1:1000) |
| Antibody | Anti-p-JNK (1/2) (rabbit monoclonal) | Cell Signaling | Cat#4668S  RRID: AB_823588 | WB (1:1000) |
| Antibody | Anti-JNK (1/2) (rabbit monoclonal) | Cell Signaling | Cat#9252S  RRID: AB_2250373 | WB (1:1000) |
| Antibody | Anti-β-actin (mouse monoclonal) | Sigma-Aldrich | Cat#A5316  RRID: AB_476743 | WB (1:5000) |
| Other | DAPI | Sigma-Aldrich | Cat#F6057 |  |
| Antibody | Alexa Fluor™ 488-GS-IB4 | Invitrogen | Cat#I21411  RRID: AB_2314662 | IF (1:500) |
| Chemical compound, drug | M-CSF | R&D Systems | Cat#416-ML |  |
| Chemical compound, drug | Celecoxib | Cayman Chemical Company | Cat#169590-42-5 |  |
| Chemical compound, drug | PGE2 | Cayman Chemical Company | Cat#14010 |  |
| Peptide, recombinant protein | Recombinant Human IL-10 | PeproTech | Cat#200-10 |  |
| Peptide, recombinant protein | Recombinant Murine IL-4 | PeproTech | Cat#214-14 |  |
| Chemical compound, drug | LPS | Sigma-Aldrich | Cat#L4391 |  |
| Chemical compound, drug | Butaprost | Cayman Chemical Company | Cat#13740 |  |
| Chemical compound, drug | Sulprostone | Cayman Chemical Company | Cat#14765 |  |
| Chemical compound, drug | Cay10598 | Cayman Chemical Company | Cat#13281 |  |
| Chemical compound, drug | H-89 | Cayman Chemical Company | Cat#10010556 |  |
| Chemical compound, drug | LY294002 | Cayman Chemical Company | Cat#70920 |  |
| Chemical compound, drug | Alexa Fluor 555 anti-rabbit IgG (H+L) (Donkey polyclonal) | Thermo Fisher Scientific | Cat#A-31572  AB_162543 |  |
| Chemical compound, drug | Alexa Fluor 555 anti-mouse IgG (H+L) (Donkey polyclonal) | Thermo Fisher Scientific | Cat#A-31570  AB_2536180 |  |
| Chemical compound, drug | Alexa Fluor 488 anti-rabbit IgG (H+L) (Donkey polyclonal) | Thermo Fisher Scientific | Cat#A21206  AB_2535792 |  |
| Chemical compound, drug | Alexa Fluor 488 anti-mouse IgG (H+L) (Donkey polyclonal) | Thermo Fisher Scientific | Cat#A21202  RRID: AB_141607 |  |
| Commercial assay or kit | Mouse PGE2 ELISA Kit | CAYMAN CHEMICAL COMPANY | Cat#514010 |  |
| Commercial assay or kit | Mouse IL10 ELISA Kit | CUSABIO | Cat#CSB-E04594m |  |
| Commercial assay or kit | Dual-Luciferase® Reporter (DLR™) Assay System | Promega | Cat#E1910 |  |
| Commercial assay or kit | Light Shift Chemiluminescent EMSA kit | Thermo Fisher Scientific | Cat#20148 |  |
| Sequence based reagent | mIL10_F | This paper | PCR primers | gctcttactgactggcatgag |
| Sequence based reagent | mIL10_R | This paper | PCR primers | cgcagctctaggagcatgtg |
| Sequence based reagent | mArg1_F | This paper | PCR primers | ctccaagccaaagtccttagag |
| Sequence based reagent | mArg1_R | This paper | PCR primers | aggagctgtcattagggacatc |
| Sequence based reagent | mYM1_F | This paper | PCR primers | agaagggagtttcaaacctggt |
| Sequence based reagent | mYM1_R | This paper | PCR primers | gtcttgctcatgtgtgtaagtga |
| Sequence based reagent | mMgl1_F | This paper | PCR primers | tgagaaaggctttaagaactggg |
| Sequence based reagent | mMgl1_R | This paper | PCR primers | gaccacctgtagtgatgtggg |
| Sequence based reagent | mMrc2_F | This paper | PCR primers | tacagctccacgctatggatt |
| Sequence based reagent | mMrc2_R | This paper | PCR primers | cactctcccagttgaggtact |
| Sequence based reagent | mTNFα_F | This paper | PCR primers | acggcatggatctcaaagac |
| Sequence based reagent | mTNFα_R | This paper | PCR primers | agatagcaaatcggctgacg |
| Sequence based reagent | mINOS_F | This paper | PCR primers | cccttcaatggttggtacatgg |
| Sequence based reagent | mINOS_R | This paper | PCR primers | acattgatctccgtgacagcc |
| Sequence based reagent | mCD16_F | This paper | PCR primers | tttggacacccagatgtttcag |
| Sequence based reagent | mCD16_R | This paper | PCR primers | gtcttccttgagcacctggatc |
| Sequence based reagent | mCD32_F | This paper | PCR primers | aatcctgccgttcctactgatc |
| Sequence based reagent | mCD32_R | This paper | PCR primers | gtgtcaccgtgtcttccttgag |
